# Knowledge, attitudes, and worries among different health literacy groups before receiving first invitation to colorectal cancer screening: Cross-sectional study

**DOI:** 10.1016/j.pmedr.2019.100876

**Published:** 2019-04-25

**Authors:** Pernille Gabel, Mette Bach Larsen, Adrian Edwards, Pia Kirkegaard, Berit Andersen

**Affiliations:** a Department of Public Health Programmes, Randers Regional Hospital, Denmark; b Department of Clinical Medicine, Aarhus University, Denmark; c Division of Population Medicine, School of Medicine, Cardiff University, UK

**Keywords:** Colorectal neoplasms, Mass screening, Health literacy, Educational status, Health knowledge, attitudes, practice, Patient participation, Socioeconomic factors, Cross-sectional studies

## Abstract

**Background:**

Colorectal cancer screening uptake is associated with knowledge, attitudes and worries about screening. People with higher levels of health literacy usually have higher screening-related knowledge, but its association with attitudes and worries is sparsely described.

The aim of this study was to describe knowledge, attitudes, and worries about colorectal cancer screening among unscreened citizens, and to estimate the association between these and health literacy.

**Methods:**

In a cross-sectional study 10,030 53–74 year-old Central Denmark Region citizens received a questionnaire assessing knowledge, attitudes, worry and health literacy. Socioeconomic and –demographic data were linked from Statistics Denmark after data collection.

**Results:**

In total, 7142 (71.2%) questionnaires were completed. A good general level of knowledge was observed (4.91 and 5.13 out of 7 for men and women, respectively). Citizens tended to be positive towards screening (21.4 and 21.3 on a 4–28 range scale for men and women respectively), and showed low levels of worries (8.8 and 9.09 on a 3–15 range scale for men and women respectively). Knowledge decreased and worries increased with lower levels of health literacy. Further, attitudes tended to be more positive with higher levels of health literacy.

**Conclusions:**

In general, citizens tend to have good knowledge, positive attitudes and few worries about colorectal cancer screening. People with lower health literacy could benefit from targeted interventions that address knowledge and worries about screening to support informed decision making.

## Highlights

• The general unscreened population has good colorectal cancer screening knowledge.
• Positive attitudes towards screening were observed among unscreened citizens.
• The general unscreened population has few colorectal cancer screening worries.
• Knowledge decreases and worries increase with lower levels of health literacy.
• Targeted interventions could support informed decision making.

## 1 Background

Colorectal cancer (CRC) is one of the three most prevalent cancer types and one of the four most frequent causes of cancer deaths among both men and women worldwide. (Ferlay et al., 2013) CRC screening using fecal occult blood tests reduces CRC mortality. (Hewitson et al., 2008) However, program effectiveness is dependent on individuals' decisions to actually take up screening.

Knowledge, attitudes, and worries about CRC and CRC screening each influence the decision to take up screening. (Jimbo et al., 2017; McCaffery et al., 2003) In general, women are more aware of screening, and have more positive attitudes towards it than men. (Janda et al., 2002; Kim et al., 1998; McCaffery et al., 2003) However, specific knowledge about CRC and CRC screening has been sparsely described among both men and women in a general unscreened population.

Health literacy is the ability to access, understand, appraise, and apply health care information in order to maintain or improve health. (Sorensen et al., 2012) CRC screening awareness tends to increase with higher levels of health literacy. (Guerra et al., 2005; Miller et al., 2007) However, the existing studies are small and only one study shows a significant association. (Miller et al., 2007) Increasing levels of detailed CRC screening knowledge may be associated with higher levels of health literacy. The tendency is not statistically significant, though, and studies differ in size from 100 to 800 study participants, and different screening histories. (Brittain et al., 2016; Essink-Bot et al., 2016; Peterson et al., 2007). Further, associations between attitudes and worries are sparsely described. Attitudes seem to be increasingly negative with lower socioeconomic status (Cullati et al., 2009; Denters et al., 2013; McCaffery et al., 2003), but the relationships between health literacy and attitudes or worries has been little studied.

When introducing CRC screening, a detailed understanding of the general population's knowledge and worries about CRC and CRC screening and their attitudes towards CRC screening is needed to better understand motivations and opportunities for screening uptake. How these factors are associated with health literacy may be important in order to target interventions for groups with lower uptake rates.

The aim of this study was to describe knowledge, attitudes, and worries towards CRC screening in citizens eligible to but not yet invited for screening. We also sought to assess the associations between these factors and levels of health literacy.

## 2 Methods

### 2.1 Setting

Biennial fecal immunochemical test (FIT) screening is offered to all Danish citizens aged 50–74 years. Individual invitations include an invitation letter and a screening kit for home-based self-sampling (collection tube, instructions, and a return envelope). If blood is detected in the stool-sample, the citizen is offered a colonoscopy. If a stool sample is not returned within 45 days after receiving the screening invitation, a screening reminder is sent to the citizen. Reminders and screening results are sent to the citizens using digital mail.

The screening program was implemented nationally over a 4-year period beginning in 2014. During this period, all individuals in the target population were invited once. Individuals aged 50–74 years on January 1st 2014 were invited according to their month of birth. A computer-generated randomized order of the birth months was created May 3rd 2013 using Stata 11.2 (STATA Copr., College Station, Tex, USA), to ensure a random order of invitation. Individuals turning 50 during the prevalence round were invited just before their birthday as were individuals turning 75 years, if they had not been invited earlier. Upon implementation of CRC screening in 2014, a national media campaign was launched to increase awareness of the screening program. However, during this study period, no campaigns were present.

The present study was carried out in the Central Denmark Region which is the second largest region in Denmark, comprising both rural and urban areas and a diverse population. The region has 1.3 million inhabitants, approximately 23% of the total Danish population. (Statistics Denmark, 2018a)

All communication from Danish authorities occurs using a secure electronic mailing platform, accessed via a digital signature, which all citizens 15 years or older are obliged to order. (The Danish Agency for Digitisation, 2017b) Older or disabled citizens can be exempt from digital communication and will receive postal mail from the authorities. As of December 2017, 7.9% of 45–74-year-old citizens are exempt from mandatory digital communication, and the proportion is decreasing. (The Danish Agency for Digitisation, 2017a) Hence, most Danish citizens are accustomed to using digital communication and receiving digital health care information.

### 2.2 Study design and population

The study was designed as a cross-sectional study among a random sample of 10,030 53–74 year-old citizens born in December and resident in the Central Denmark Region at August 8th 2017. Citizens born in December were scheduled to be invited to CRC screening from October to December 2017 via weekly distributed screening invitations. Hence, all included citizens were yet to be invited for CRC screening for the first time. Citizens 50–52 years of age had already received an invitation for CRC screening by August 2017 and were excluded from the study population. Citizens were informed about the study via digital information letters. No media campaign or coverage of the study was conducted.

### 2.3 Questionnaire data

Data were collected via electronic questionnaires assessing four outcomes using four scales. The four scales of the questionnaire were pilot tested among 79 50–74-year-old citizens in the Central Denmark Region before data collection took place.

Knowledge about CRC and CRC screening was assessed by seven items (four on CRC and three on CRC screening) developed by the authors and formulated as statements to which citizens could answer correct/incorrect/don't know. This type of scale has previously been used when assessing knowledge about CRC and CRC screening. (Denters et al., 2015, Halley et al., 2015, Lewis et al., 2010, Lindblom et al., 2012, Steckelberg et al., 2011) The scale was validated among 79 citizens. Factor analysis confirmed a unidimensional scale with an acceptable internal consistency (Cronbach's α: 0.58). All response categories were represented, and no floor or ceiling effects were observed. The scale was considered relevant and comprehensible by the citizens, and few missing items were observed, pointing towards a valid scale. Each correct answer was scored one point. Incorrect answers, don't know and missing values were scored zero. Scale scores ranged from 0 to 7. Higher scores indicated greater knowledge.

CRC worry was assessed in three items formulated as statements to which citizens answered on a five-point Likert-like scale (disagree/disagree somewhat/neither agree nor disagree/agree somewhat/agree). This assessment has been previously used to assess cancer worries. (Hay et al., 2005; Sutton et al., 1994) Each item scored 1–5 points, yielding a total score of 3–15 points. Higher scores indicated more worry. In cases with missing items, the total scale score was coded missing. Internal consistency was good (Cronbach's α: 0.81).

The attitudes scale was adapted to CRC screening from the original version developed by Marteau et al. (Marteau et al., 2001) and translated using forward-backward translation, as described by Beaton et al. (Beaton et al., 2000) The scale had four items, formulated as statements “For me, having the screening test for colorectal cancer will be…” followed by a 7-point scale ranging from Beneficial-Harmful/Important-Unimportant/Good thing-Bad thing/Pleasant-Unpleasant. The scale ranged from “agree”, to “neutral” in the center to “agree” at the other end. Scoring ranged from 4 to 28 points. If one or more items were missing, the total score was coded missing, according to the scoring manual. (Marteau et al., 2001; Michie et al., 2002) Internal consistency was acceptable (Cronbach's α: 0.71).

Health literacy was assessed using the HLS-EU-Q16 scale. (HLS-EU Consortium, 2012) This scale was translated into Danish by another research group using forward-backward translation. (Beaton et al., 2000) Sixteen items scored zero or one point each (total score of 0–16 points). The scores 0–8 corresponded to “inadequate” health literacy, while 9–12 corresponded to “problematic” health literacy, and 13–16 corresponded to “adequate” health literacy. Missing items were scored 0, and only two or fewer missing items were accepted, otherwise the total score was coded missing, according to the manual. (Sørensen et al., 2015) Internal consistency of the scale was good (Cronbach's α: 0.88).

### 2.4 Background data

Background data were obtained from Statistics Denmark (Statistics Denmark, 2016) for respondents and non-respondents. According to the classification defined by Statistics Denmark, ethnicity was categorized into native Danes, Western immigrants (EU, Andorra, Australia, Canada, Iceland, Lichtenstein, Monaco, New Zealand, Norway, San Marino, Switzerland, USA, and the Vatican state) and non-Western immigrants (others). (Statistics Denmark, 2017) Marital status was dichotomized into married/cohabitant or living alone. Income was categorized into three groups according to tertiles of the specific dataset. A weighted personal income was used, based on the OECD-modified equivalence scale adjusting the total household income according to the number of family members in the household. (Statistics Denmark, 2018b) Education was categorized into three groups according to ISCED 2011: lower educational attainment (≤10 years of education; level 1–2), medium educational attainment (10–15 years of education; level 3–5), and higher educational attainment (>15 years of education; level 6–8). (UNESCO Institute for Statistics, 2011; UNESCO, 2014) Employment was categorized into 1) self-employed/chief executive; 2) employed; 3) unemployed/welfare benefits; 4) retired; and 5) other.

### 2.5 Data collection

The random population sample was delivered by the Danish Health Data Authority from the Danish Civil Registration System. (Pedersen, 2011) The sample contained information on the unique civil registration number (CPR-number), names, and addresses.

Questionnaire data were collected using the REDCap Software (Version 6.12.0 - © 2016 Vanderbilt University). (Harris et al., 2009) During August 2017 unique personal links for the 30 item web-based questionnaire were provided to all included citizens via digital mail. Non-respondents received a digital reminder after two weeks. Lastly, a phone call was made to non-respondents after four weeks, offering to fill out the questionnaire via the telephone. Telephone calls were made by an external research and insights management solutions company. (Epinion, 2017) Questionnaire data collection was terminated in September 2017.

Survey data were linked with registry-based background data from Statistics Denmark in December 2017 using the individual CPR-number.

### 2.6 Statistical analyses

Descriptive presentation of data and comparison between groups were done using percentages, proportions and chi^2^-tests for categorical outcomes, and mean and Student's *t*-test for continuous outcomes.

The association between knowledge, attitude, and worry scores and health literacy was assessed using linear regression analyses, estimated as mean difference in scores. Analyses were stratified by gender, and adjustment was done for age, ethnicity, marital status, and educational attainment. Both crude and adjusted estimates are presented.

Statistical analyses were performed on a 5% significance level in Stata/SE 15 (STATACorp LP, College Station, Texas, USA). Estimates are presented with 95% confidence intervals (95% CI).

### 2.7 Study approvals

Collection and obtaining survey data were permitted by the Danish Data Protection Agency (J.no.: 2012-58-006/Case no.: 1-16-02-94-16). Clearance for data collection was obtained from the Danish Patient Safety Authorities (J.no.: 3-313-1729-1) and the Central Denmark Region Committee on Health Research Ethics (143/2016). Study subjects consented to study participation by submitting the questionnaire.

## 3 Results

A total of 7142 people completed the questionnaire (response rate of 71.2%). Respondents were more often married or cohabitant, with medium education levels, employed, with medium or high incomes and ethnic Danes than non-respondents (Table 1). The distributions of frequencies between respondents and non-respondents were statistically significantly different in all variables.

**Table 1:** Demographic characteristics of survey respondents and non-respondents.

	Respondents (*n* = 7142 71%)	Non-respondents (*n* = 2888 29%)	Total population (*n* = 10,030)
	N (%)^a^	N (%)^a^	N (%)^a^
Gender			
Male	3316 (46)	1426 (49)	4742 (47)
Female	3826 (54)	1462 (51)	5288 (53)
Age			
Mean (CI)	63.5 (63.3;63.7)	63.9 (63.7;64.2)	63,6 (63.5;63.8)
53–59	2474 (35)	997 (35)	3471 (35)
60–64	1742 (24)	614 (21)	2356 (23)
65–69	1568 (22)	623 (22)	2191 (22)
70–74	1358 (19)	654 (23)	2012 (20)
Ethnicity			
Danish	6854 (96)	2611 (91)	9465 (95)
Western immigrant	159 (2)	87 (3)	246 (3)
Non-Western immigrant	122 (2)	183 (6)	305 (3)
Marital status			
Married/cohabitant	5484 (77)	1689 (59)	7173 (72)
Single	1651 (23)	1192 (41)	2843 (28)
Income			
< €30,000	1955 (27)	1401 (49)	3356 (33)
€30,000–€43,000	2406 (34)	779 (27)	3185 (32)
≥ €43,000	2781 (39)	708 (25)	3489 (35)
Education			
≤ 10 years	1679 (24)	1023 (37)	2702 (27)
10–15 years	4849 (69)	1597 (57)	6446 (65)
> 15 years	530 (6)	181 (6)	711 (7)
Occupation			
Self-employed/Chief executive	498 (7)	182 (6)	680 (7)
Employed	3135 (44)	903 (31)	4038 (40)
Not employed/welfare benefits	230 (3)	155 (5)	385 (4)
Retired	3194 (45)	1579 (55)	4773 (48)
Other	82 (1)	66 (2)	148 (1)

A statistically significant difference (*p* < 0.01) within all groups was observed using chi^2^ (categorical) or student's T-test (continuous).

a Some column sums do not add up due to missing values, and some percentages do not add up to 100 because of roundings.

Women had a slightly, but statistically significantly higher mean knowledge score than men (scale score 5.16 (95% confidence interval (CI) 5.11 to 5.21) and 4.93 (4.87 to 4.99), respectively). In the items regarding CRC symptoms and screening the proportion of correct answers ranged from 79% to 90% in men and women. The items less frequently answered correctly (36% to 54%) were about bowel cancer triggers and bowel cancer incidence (Table 2).

**Table 2:** Knowledge, screening attitudes, worries, and health literacy among respondents.

	Respondents (n = 7142)	
	Male (*n* = 3316) Mean (CI)	Female (*n* = 3826) Mean (CI)
Knowledge^a^		
Scale score (0–7)	4.93 (4.87;4.99)	5.16 (5.11;5.21)
1) Colorectal cancer is often triggered by a scratch in the bowel (incorrect)^b^	44.9% (43.2;46.6)	53.9% (52.3;55.5)
2) 1 out of 20–25 people will be diagnosed with colorectal cancer before the age of 75 years (correct)^b^	42.4% (40.7;44.1)	36.5% (35.0;38.1)
3) It is possible to have an undetected colorectal cancer for a longer period of time without having any symptoms (correct)^b^	80.9% (79.5;82.2)	85.0% (83.8;86.1)
4) Colorectal cancer screening is for symptomatic people only (incorrect)^b^	82.6% (81.3;83.8)	85.2% (84.0;86.3)
5) You will have to go to the doctor, if you have symptoms of colorectal cancer, although the screening result did not detect any blood in the stool (correct)^b^	85.3% (84.1;86.5)	89.9% (88.9;90.8)
6) Blood in the stool is an undeniable sign of colorectal cancer (incorrect)^b^	78.2% (76.8;79.6)	82.5% (81.3;83.7)
7) Abdominal pain and altered bowel habits may be symptoms of colorectal cancer (correct)^b^	79.4% (78.0;80.7)	84.3% (83.2;85.5)
Attitudes^c^		
Scale score (4–28)	21.4 (21.2;21.6)	21.3 (21.2;21.5)
1) Harmful (1) – Beneficial (7)	5.97 (5.93;6.02)	5.95 (5.90;5.99)
2) Unimportant (1) – Important (7)	5.78 (5.73;5.83)	5.82 (5.77;5.87)
3) Bad thing (1) – Good thing (7)	5.60 (5.54;5.65)	5.65 (5.60;5.70)
4) Unpleasant (1) – Pleasant (7)	4.06 (4.01;4.12)	3.87 (3.81;3.92)
Worries^d^		
Scale score (3–15)	8.80 (8.70;8.89)	9.09 (9.00;9.17)
1) I get worried when I think about colorectal cancer (1–5)	3.56 (3.32;3.40)	3.44 (3.41;3.48)
2) I get scared when I think about colorectal cancer (1–5)	3.05 (3.02;3.09)	3.21 (3.18;3.25)
3) I am concerned that colorectal cancer is detected if I participate in screening (1–5)	2.43 (2.39;2.47)	2.50 (2.46;2.53)
Health literacy^e^		
Scale score (0–16)	11.6 (11.5;11.7)	12.1 (12.0;12.2)
Adequate (n(%))	1441 (44.8)	1862 (50.4)
Problematic (n(%))	1191 (37.1)	1327 (35.9)
Inadequate (n(%))	583 (18.1)	505 (13.7)

a Knowledge: Individual items are formulated as statements. Respondents mark if the statement is “correct” or “incorrect”. Single items are scored 0–1 points (range: 0–7).

b The proportion who correctly marked the item.

c Attitudes: Single items are scored 1–7 points. Scale score ranges from 4 to 28 points. Higher values indicate more positive attitudes. If one or more items are missing, attitudes score is coded as missing.

d Worries: Item score range: 1–5; scale score range: 3–15; Higher scores indicate higher levels of worry.

e Health literacy: Scale score range: 0–16. Adequate health literacy: 13–16; Problematic health literacy: 9–12; Inadequate health literacy: 0–8.

Table 2 also shows that overall both men and women were equally positive towards CRC screening in general (scale scores 21.4 (CI 21.2 to 21.6) and 21.3 (CI 21.2 to 21.5) respectively). They tended to be more neutral regarding whether screening is pleasant or unpleasant, even though women found it more unpleasant than men. Further, women assessed themselves slightly more worried than men (scale score 9.09 (CI 9.00 to 9.17) and 8.80 (CI 8.70 to 8.89) respectively). Lastly, health literacy was adequate in 44.8% of men and 50.4% of women and inadequate in 18.1% of men and 13.7% of women (Table 2).

The adjusted regression analyses showed that the level of knowledge decreased and the level of worries increased for each level decrease in health literacy. Hence, knowledge scores were statistically significantly lower among men and women with inadequate health literacy as compared to men and women with adequate health literacy (mean differences: −0.44 (CI −0.61 to −0.28) and −0.58 (CI −0.72 to −0.43) out of 7, respectively). Likewise, a lower knowledge score was observed among women with problematic health literacy as compared to women with adequate health literacy (mean difference: −0.16 (CI −0.26 to −0.06)). Further, a statistically significantly higher level of worry was observed when comparing adequate health literacy to problematic and inadequate health literacy (among problematic health literacy level men: 0.5 (CI 0.2 to 0.7) and women: 0.5 (CI 0.3 to 0.7); and inadequate health literacy level men: 1.0 (CI 0.8 to 1.3) and women: 1.1 (CI 0.8 to 1.4)). In the adjusted analysis, attitudes showed a non-significant dose-response pattern with higher levels of health literacy being slightly associated with a more positive attitude (Table 3).

**Table 3:** Knowledge, attitudes and worries among respondents with different levels of health literacy.

Health literacy	Knowledge^a^			Attitudes^a^			Worry^a^		
	Score	Mean dif (CI)		Score	Mean dif (CI)		Score	Mean dif (CI)	
	Mean (CI)	Crude	Adjusted^b^	Mean (CI)	Crude	Adjusted^b^	Mean (CI)	Crude	Adjusted^b^
Male									
Adequate	5.04 (4.95;5.13)	0 (ref)	0 (ref)	21.6 (21.3;21.8)	0 (ref)	0 (ref)	8.45 (8.31;8.59)	0 (ref)	0 (ref)
Problematic	5.00 (4.91;5.10)	−0.03 (−0–16;0.10)	−0.00 (−0.13;0.13)	21.4 (21.1;21.7)	−0.20 (−0.55;0.15)	−0.22 (−0.58;0.13)	8.91 (8.75;9.06)	**0.5 (0.3;0.7)**	**0.5 (0.2;0.7)**
Inadequate	4.51 (4.36;4.67)	**−0.53 (−0.69;−0.36)**	**−0.44 (−0.61;−0.28)**	21.2 (20.8;21.5)	−0.43 (−0.87;0.01)	−0.37 (−0.81;0.07)	9.54 (9.32;9.76)	**1.1 (0.9;1.4)**	**1.0 (0.8;1.3)**
Female									
Adequate	5.33 (5.27;5.40)	0 (ref)	0 (ref)	21.5 (21.2;21.7)	0 (ref)	0 (ref)	8.77 (8.64;8.89)	0 (ref)	0 (ref)
Problematic	5.13 (5.05;5.21)	**−0.19 (−0.31;−0.10)**	**−0.16 (−0.26;−0.06)**	21.3 (21.0;21.5)	−0.20 (−0.53;0.14)	−0.15 (−0.48;0.19)	9.28 (9.14;9.42)	**0.5 (0.3;0.7)**	**0.5 (0.3;0.7)**
Inadequate	4.57 (4.42;4.71)	**−0.75 (−0.89;−0.60)**	**−0.58 (−0.72;−0.43)**	20.9 (20.5;21.3)	**−0.57 (−1.04;−0.10)**	−0.40 (−0.88;0.07)	9.95 (9.71;10.20)	**1.2 (0.9;1.4)**	**1.1 (0.8;1.4)**

a Linear regression analysis, estimates in bold types are statistically significantly different from 0 (*p* < 0.05).

b Adjusted for age, ethnicity, marital status, and educational attainment.

## 4 Discussion

### 4.1 Main findings

In this cross-sectional study among individuals from the general population eligible but not yet invited to the Danish CRC screening program, we found that both men and women had generally good levels of knowledge, tended to be positive towards screening, and had low levels of worries. Women tended to be more knowledgeable and more worried than men. Individuals with lower health literacy tended to have less CRC and CRC screening knowledge and to be more worried than individuals with higher health literacy. Attitudes towards cancer screening showed a similar but less pronounced association and not reaching statistical or clinical significance. Inadequate health literacy was observed in one sixth of the population, and one third had problematic health literacy levels.

### 4.2 Strengths and limitations

A major strength of this study is the response rate of 71.2%. Using digital mail with an easy link to answer the questionnaire via the internet, and using phone calls instead of a second digital reminder to target especially those exempt from digital communication, may have contributed to the high response rate. However, non-respondents still differed from respondents. If citizens with lower knowledge and increased worries tend to be non-respondents, there is a risk of selection bias, resulting in an underestimation of the association between health literacy and knowledge/worries.

Using registry data of high validity and few missing values in combination with validated scales, accompanied with forward/backward translation of scales in foreign languages and piloting of the questionnaire in the target population, contributed to a low risk of information bias in exposure, outcome, and background measures. Health literacy was measured using a validated scale. It has been suggested to use continuous scales to measure subjective values, since the arbitrary cut-offs defined in these scales may appear to indicate that a true cut-off exists, rather than reflecting a continuous spectrum of the truth. (Ghanouni et al., 2016) Nevertheless, the scale has been developed and validated using these cut-offs (Sørensen et al., 2015), and hence, this approach was used to facilitate comparison with other studies. Social desirability bias cannot be ruled out for attitudes questions. (Fisher, 1993) However, the self-administered design minimizes this risk.

The population-based design, inviting a random sample of the population, supports representativeness. The results from this study may be generalized to and useful in comparable communities considering implementation of CRC screening programs.

### 4.3 Comparison to other studies

CRC screening attitudes were not associated with health literacy in adjusted analyses. This observation confirms a previous study (Essink-Bot et al., 2016), but is emphasized now due to greater power and generalizability in the present study.

Knowledge regarding the triggers and the incidence of CRC was generally low. Nevertheless, most respondents were aware that screening is aimed at non-symptomatic citizens, that symptoms might be non-specific and present late, and that one should seek medical advice in case of symptoms. There was an acceptable general understanding of the concept of CRC screening, and hence a good prerequisite for making informed decisions about CRC screening uptake. This is consistent with the findings of Forbes et al., who observed that Danish citizens aged 50 years or older were more likely to agree that cancer causes morbidity, impairment and death, as compared to citizens from other European countries and Australia. (Forbes et al., 2013)

In general the attitudes scores were favorable towards screening. This is consistent with previous findings of a rate of favorable attitudes of 71.8% among US citizens not previously screened. (Brenner et al., 2016) A direct comparison is difficult however, since the attitudes data in the US study were dichotomized, and further, the study was conducted among 16–74 year old citizens. Our data did not support previous findings that women are more positive towards screening than men. (McCaffery et al., 2003) As another Danish study showed that women take up colorectal cancer screening more than men (Larsen et al., 2017), this indicates that other factors than attitudes also play a role in actual screening behavior.

Lower levels of health literacy are associated with lower self-efficacy in regards to CRC screening. (von Wagner et al., 2009) We observed 18% and 14% of men and women, respectively, with inadequate health literacy, which was slightly higher than the 12.4% observed overall in eight European countries. (Sørensen et al., 2015) The slightly higher proportion of citizens with inadequate health literacy observed in our study population might be explained by a lower response rate and the 49-item health literacy scale used in the European study, even though the personal interviewing in that study might have increased the proportion of lower health literacy citizens who responded. (Sørensen et al., 2015) In general, men tend to have lower levels of health literacy (Brittain et al., 2016; Peterson et al., 2007; Sørensen et al., 2015), a tendency also observed in our study.

### 4.4 Implications for society

Health literacy tended to be associated with CRC screening knowledge, attitudes and worries. However, an increase from 4.51 to 5.04 in knowledge is most likely too small to consider practically or clinically relevant at an individual level. Nevertheless, it is well-known that even small shifts in population mean can lead to important health gains. (Rose, 2001)

Lower health literacy levels are associated with a tendency to only read headings and look at figures in written information material. (Fransen et al., 2017) Hence, accessible and comprehensible information materials focusing on the simplicity of performing the screening test, and the outcomes of screening, in order to support citizens' self-efficacy regarding CRC screening and enhance favorable attitudes (Ajzen, 1991) may be beneficial to both health authorities and citizens. Further, general public awareness about CRC screening might alter the perception of screening among the general population, which may also affect screening behaviors.

Lastly, research into specific interventions to increase knowledge and decrease worries about CRC and CRC screening for the general population is needed.

## 5 Conclusions

In general, citizens had good knowledge positive attitudes and few worries about CRC and CRC screening. Women tended to be more knowledgeable and more worried than men. Health literacy tended to some degree to be associated with knowledge, attitudes and worries. The association implies a limited effect at an individual level, but there is potential for beneficial effects at population level owing to an increase in means.

## Conflicts of interest

None.
